# Testing a Family Supportive End of Life Care Intervention in a Chinese Neonatal Intensive Care Unit: A Quasi-experimental Study With a Non-randomized Controlled Trial Design

**DOI:** 10.3389/fped.2022.870382

**Published:** 2022-07-22

**Authors:** Rong Zhang, Qian Tang, Li-hui Zhu, Xiao-ming Peng, Na Zhang, Yue-e Xiong, Mu-hua Chen, Ke-liang Chen, Dan Luo, Xun Li, Jos M. Latour

**Affiliations:** ^1^Department of Neonatology, Hunan Children's Hospital, Changsha, China; ^2^Department of Nursing, Hunan Children's Hospital, Changsha, China; ^3^Department of Nursing, Hunan University of Chinese Medicine, Changsha, China; ^4^Department of Clinical Research Center, Hunan Children's Hospital, Changsha, China; ^5^Faculty of Health, School of Nursing and Midwifery, University of Plymouth, Plymouth, United Kingdom

**Keywords:** neonatal death, end-of-life care, infants, parents, Neonatal Intensive Care Unit, family-centered care

## Abstract

**Background:**

Neonatal death often occurs in tertiary Neonatal Intensive Care Units (NICUs). In China, end-of-life-care (EOLC) does not always involve parents.

**Aim:**

The aim of this study is to evaluate a parent support intervention to integrate parents at the end of life of their infant in the NICU.

**Methods:**

A quasi-experimental study using a non-randomized clinical trial design was conducted between May 2020 and September 2021. Participants were infants in an EOLC pathway in the NICU and their parents. Parents were allocated into a family supportive EOLC intervention group or a standard EOLC group based on their wishes. The primary outcomes depression (Edinburgh Postnatal Depression Scale for mothers; Hamilton Depression rating scale for fathers) and Satisfaction with Care were measured 1 week after infants' death. Student *t*-test for continuous variables and the Chi-square test categorical variables were used in the statistical analysis.

**Results:**

In the study period, 62 infants died and 45 infants and 90 parents were enrolled; intervention group 20 infants, standard EOLC group 25 infants. The most common causes of death in both groups were congenital abnormalities (*n* = 20, 44%). Mean gestational age of infants between the family supportive EOLC group and standard EOLC group was 31.45 vs. 33.8 weeks (*p* = 0.234). Parents between both groups did not differ in terms of age, delivery of infant, and economic status. In the family support group, higher education levels were observed among mother (*p* = 0.026) and fathers (*p* = 0.020). Both mothers and fathers in the family supportive EOLC group had less depression compared to the standard EOLC groups; mothers (mean 6.90 vs. 7.56; *p* = 0.017) and fathers (mean 20.7 vs. 23.1; *p* < 0.001). Parents reported higher satisfaction in the family supportive EOLC group (mean 88.9 vs. 86.6; *p* < 0.001).

**Conclusions:**

Supporting parents in EOLC in Chinese NICUs might decreased their depression and increase satisfaction after the death of their infant. Future research needs to focus on long-term effects and expand on larger populations with different cultural backgrounds.

**Clinical Trial Registration:**

www.ClinicalTrials.gov, identifier: NCT05270915.

## Introduction

In 2019, the World Health Organization (WHO) reported that neonatal death within the first 28 days of life reached 17 per 1,000 live births, estimating around 2.4 million neonates (1). In China, neonatal death was 3.5 per 1,000 live births in 2019, which was around 57,000 deaths (2). End-of-life care (EOLC) has been emphasized by the WHO Global Action Plan 2013–2020 (3, 4). The mandate by the WHO highlights the need for improvement in infant's EOLC and the support of parents and family in the Neonatal Intensive Care Unit (NICU).

From a historical perspective, the care around death of neonates was first addressed in the United States in the 1980's. In 1982, Silverman described that EOLC has been successfully implemented in hospice settings for newborns (5). Over the years, EOLC has been further progressed in European countries and Northern America leading to a number of national guidelines and clinical practice recommendations (6–8). And recently, palliative care has become a new service in many healthcare settings and EOLC can play an important part in palliative care.

Recent studies have focused on EOLC decisions (9), pain and comfort management (10) and implementation of the palliative care sub-specialty within Neonatology (11). Unfortunately, EOLC received less attention in Asia, specifically in mainland China (12). A literature review investigating the EOLC practices in Asian countries identified only 11 empirical studies from Hong Kong, India, Israel, Japan, Mongolia, Taiwan, and Turkey (13). Studies around EOLC from Taiwan explored the attitudes of NICU staff and identified a number of barriers in delivering high quality of EOLC (14, 15). The most common barriers were insufficient training in communication with parents, staffing shortages and lack of unit policies in supporting palliative care. Compared to European countries and Unite States, less evidence is available from Asian countries in how parents are involved in the care of their infant and specifically how family-centered care (FCC) is included in EOLC.

Since 2010, FCC has gained more attention in China and has been gradually implemented in Chinese NICUs. An FCC program was implemented in our NICU department at Hunan Children's Hospital in Changsha China, and contributed to a wider implementation across Chinese NICUs (16–19). Three trials were conducted to test FCC interventions related to parental empowerment (training of parents and participation of parents in the care of their infant) demonstrating significant improvements in breastfeeding and quality of life. The studies also documented a decrease in parental anxiety and depression as well as an improvement in parent satisfaction (16–18). Despite different beliefs, cultures, attitudes and policy, the EOLC remains unexplored in China without rigorous evidence of supporting parents in end-of-life decisions and care. As parental support is an important component of FCC, the support of parents during EOLC has different perspectives and needs different approaches. However, one cannot deliver poor EOLC while providing excellent FCC. Both practices are interlinked. Therefore, our NICU is translating and implementing FCC into EOLC practices.

In mainland China, parents are mostly the main decision-makers in withdrawing life-sustaining treatments in infants and neonatologists often follow the wishes of the parents. However, there is limited experience in supporting parents after the decision is made to withdraw treatment. Therefore, the aim of this study was to develop a family supportive EOLC intervention and to evaluate parent reported outcome measures related to depression and satisfaction.

## Materials and Methods

This quasi-experimental study adopted a non-randomized controlled trial (non-RCT) design because blinding was not possibly due to the nature and delivery of the intervention. The study was registered in clinicalTrials.gov (approval number NCT05270915). The study was conducted between 6^th^ of May 2020 and 20^th^ of September 2021. The guideline ‘Evaluating complex interventions in end of life care: the MORECare statement on good practice generated by a synthesis of transparent expert consultations and systematic reviews' was used to report this study (20).

### Setting

The study setting was the tertiary NICU at the stand-alone Hunan Children's Hospital in Changsha, China. The 180-bed NICU department serves as a regional tertiary center for all infants above 24 weeks gestational age requiring intensive care treatment. Main causes of mortality in our NICU are congenital malformation, preterm birth and septic shock. In 2020 and 2021, the annual NICU admission rate was around 4,000 infants. The annual mortality rate of the NICU in the past 5 years was between 3 and 5%. Since the introduction of FCC in our NICU, parents are allowed to visit the NICU in daytime (8.00–17.30 h) and participate in basic care of their infant and are supported by medical and nursing staff (17, 20).

### Patient and Public Involvement and Engagement

Before the study protocol was finalized, we organized a patient and public involvement and engagement meeting with 15 parent couples with previous experience in neonatology. The individual conversations with both mothers and fathers of 15 infants were focused on the proposed study methods, intervention, and outcome measures. Overall, most parents thought that their involvement in EOLC was important to reduce depression during and after the death of their infant. Parents indicated that they would value the support of NICU staff and would welcome a separate room to stay with their baby in the final days of life. Most parents also suggested having a psychologist in the NICU team and having their support at the EOLC. In terms of follow-up, most parents indicated that they did not want a long-term follow-up meeting or complete surveys 1 month after NICU discharge. The suggestions of the parents were amended in the final study protocol.

### Study Participants and Recruitment

Inclusion criteria were infants whose treatment was withdrawn at Corrected Gestational Age (CGA) <28 days and their parents. The exclusion criteria were infants with an expected time of death <3 h after NICU admission. Parents were excluded if they had mental illness or language issues that might limit their integration and communication with the healthcare team.

After an end-of-life decision was made, a research nurse informed the parents about the study. Participation was based on the parents' decision and after written consent. The allocation of the infant and parents to the intervention or control group was case-controlled based on the wishes of the parents. If parents wanted to stay in the NICU with their infant during the EOLC pathway, parents were allocated to the intervention group. If parents did not want to stay in the NICU during the EOLC pathway, their infant would stay in our NICU and receive standard EOLC care.

### Standard Care and Intervention

The standard EOLC included the international guidance of palliative care and EOLC in neonatology (21–23). In China, parents are often the decision-makers of their infant's treatment and the NICU clinicians usually respect the parent's decision (24). After parents have decided to withdraw treatment, standard EOLC is initiated and includes monitoring of vital signs and withholding or withdrawing rescue procedures such as intubation and intravenous infusion. Unnecessary lines are removed and pain management is provided by analgesia. Comfort care is provided by nurses including basic care such as skin care and oral care. After the infant died, the NICU physician informs the parents by phone.

The intervention “family supportive EOLC” was developed based on the international guidelines of family-centered care (25) with additional aspects of care and support. We designed a separated single-bedded EOLC room for the infant and parents. Other family members, such as grandparents or siblings, were allowed to visit the infant and parents. The design of the room included the option for parents to stay comfortably on a sofa to relax and to play soothing music. Parents were encouraged to stay as long as they want and participate in basic care including physical contact with their infant. The nurses supported the parents in creating commemorative items such as a “Yuan man” box with photos, baby handprint cards, footprint cards, a lock of hair and other precious memory items. A psychologist, in collaboration with our NICU, and a neonatologist supported the parents by individual interviews on a daily basis to listen to the concerns of parents and to provide emotional support. To ensure consistency in delivering the intervention, the medical staff and psychologist were trained in delivering the interviews and EOLC practices.

### Outcomes Measures and Data Collection

The primary outcomes were depression and satisfaction as reported by parents at one week after infant's death. Because the Chinese version of the Edinburgh Postnatal Depression Scale (EPDS) has not been validated among fathers, we decided to use the Chinese version of the Hamilton Depression rating scale (HAMD) to evaluate depression among fathers. The Chinese version of the EPDS was used to assess depression among mothers (26, 27). The HAMD includes 17 items with a 3 or 5-point Likert answer option scale with a total score of 78 (28). The HAMD has been translated and validated in Chinese. The internal consistency of the Chinese version demonstrated a Cronbach's alpha of 0.646 (29). The EPDS is developed to measure the depression of mothers after NICU (30). The scale includes 10 items with a 4-point Likert answer option scale with a total score of 30. The EPDS has been translated and validated in Chinese among mothers. The internal consistency of the Chinese version has been adequate with a Cronbach's alpha of 0.76 (31).

Parent satisfaction was measured by the hospital standard parent satisfaction survey completed by both parents.The parent satisfaction with care instrument was our hospital standardized parents satisfaction with care questionnaire including 20 items using a 5-point Likert answer option scale with a total score of 100. It included 4 parts of medical treatment, medical staff's negotiation attitude, hospital settings and social service. This scale is used among all parents in our hospital on a weekly basis by an external company.

Basic parent and infant characteristics were collected from the medical charts. Infants' characteristics included prenatal history, diagnoses, on-going therapy at time of withdrawal of treatment. The parental characteristics included age, mode of delivery, education and family income. The parental outcome measures, depression and satisfaction, were collected 1 week after the death of the infants during a face-to-face follow-up meeting in the hospital with the psychologist.

### Data Analysis

The statistical software package “IBM Corp. Released 2013. IBM SPSS Statistics for Windows, Version 22.0. Armonk, NY: IBM Corp” was used for the analysis. The distribution of baseline characteristics for two groups are summarized using descriptive statistical methods. Student *t*-test for continuous variables and the Chi-square test for categorical variables were used to analyze the outcomes.

### Ethics

Ethical approval was granted by the Ethics Committee of Hunan Children's Hospital (HCHLL-2020-23). The study procedures adhered to the International Council for Harmonization and Good Clinical Practice guidance (32) and the principles of the Declaration of Helsinki (33). Parents were informed about the study objectives, written informed consent was obtained, and parents were able to withdraw from participation at any time.

## Result

**Figure 1:**
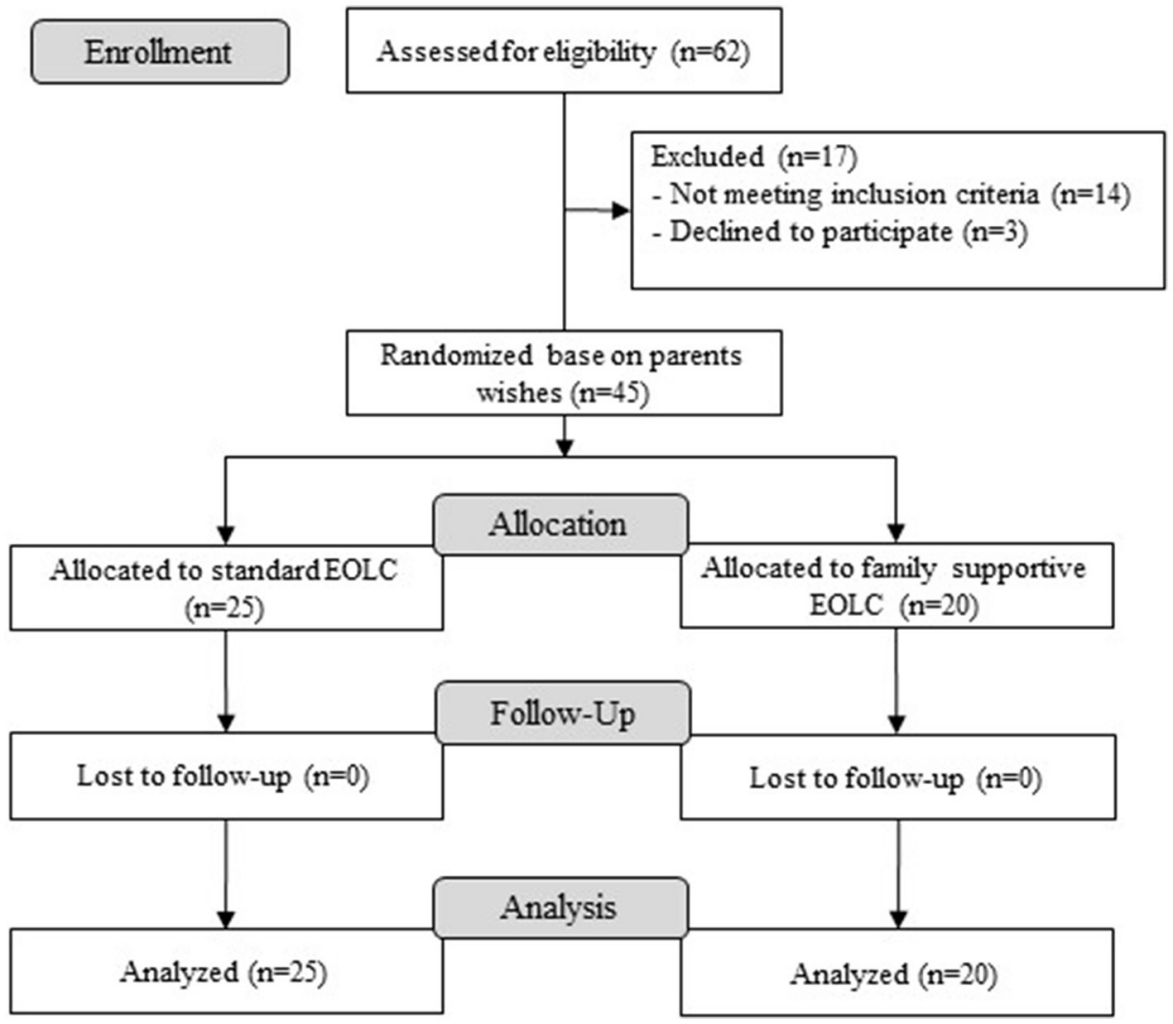
Study flow chart.

In total, 62 infants died in the NICU during the study period. Of these, 45 infants and 90 parents were screened and enrolled in the study (Figure 1). The infants' average gestational ages were smaller and birth weights were lower in the family supportive EOLC group compared with the standard care group, but no significant differences were observed (Table 1). The infants' gender did not significantly differ between both groups (male: 12 vs 16, *p* = 0.783). The most common causes of death were congenital abnormalities in both group. The median age of death in the standard care group was lower than in the family supportive EOLC group (Table 1). The main reasons of treatment withdrawal were deficiency in family financial support (not able to pay the additional hospital expenses), poor neurological prognosis and serious condition (Table 1).

**Table 1:** Infants' characteristics.

**Infants**	**Family supportive EOLC (*n* = 20)**	**Standard EOLC (*n* = 25)**	***P* value**
Gender, male; *n* (%)	12 (60)	16 (64)	0.783
Gestational age in weeks; mean (SD)	31.45 (5.18)	33.8 (5.56)	0.234
Birth weight in grams; mean (SD)	16,77 (974.2)	2,179 (1,060.3)	0.302
Length of stay in days; mean (SD)	16.7 (27.5)	16.7 (28.0)	0.828
Age at death in days; mean (SD)	30.8 (37.2)	23.9 (32.6)	0.710
Days from withdraw decision to death in days; mean (SD)	0.4 (0.68)	0.36 (1.25)	0.540
Location of infant's birth; *n* (%)			0.463
City	8 (40)	6 (24)	
Town	4 (20)	3 (12)	
Village	8 (40)	16 (64)	
Major cause of death; *n* (%)			0.913
Respiratory failure	2 (10)	1 (4)	
Congenital abnormalities	9 (45)	11 (44)	
Hypoxic-ischaemic encephalopathy	2 (10)	2 (8)	
Necrotising enterocolitis	2 (10)	2 (8)	
Prematurity	2 (10)	1 (4)	
Septic shock	1 (5)	3 (12)	
Hematology disease	1 (5)	2 (8)	
MODS	1 (5)	3 (12)	
Reason to withdraw decision; *n* (%)			0.405
Economic level	3 (15)	4 (16)	
Poor prognosis	6 (30)	12 (48)	
Infants' critical ill condition	11 (55)	9 (36)	

*EOLC, End of life care; MODS, multiply organ dysfunction syndrome; SD, Standard Deviation*.

The characteristics of the 90 parents (45 mothers and 45 fathers) are presented in Table 2. There were no differences in parent's age, way of delivery, and economic status. Both the mothers and fathers in the family supportive EOLC groups had significantly higher educational background compared to the parents in the standard EOLC group (Table 2).

**Table 2:** Parents' characteristics.

**Parents**	**Family supportive EOLC (*n* = 20)**	**Standard EOLC (*n* = 25)**	***P* value**
Mothers' age; mean (SD)	31.6 (5.41)	29.8 (5.29)	0.785
Delivery, vaginal; *n* (%)	11 (55)	12 (48)	0.641
Mother's education degree; *n* (%)			0.026*
Above university level	14^a^ (70)	8^b^ (32)	
High school level	2^a^ (10)	10^b^ (40)	
Primary school level	4^a^ (20)	7^a^ (28)	
Father's age; mean (SD)	34.5 (7.47)	31.6 (5.54)	0.818
Father's education degree; *n* (%)			0.020*
Above university level	13^a^ (65)	6^b^ (24)	
High school level	3^a^ (15)	10^a^ (40)	
Primary school level	4^a^ (20)	9^a^ (36)	
Family income level; *n* (%)			0.471
<3,000(¥)	6 (30)	4 (16)	
3,000–6,000(¥)	9 (45)	11 (44)	
>6,000(¥)	5 (25)	10 (40)	

a, bThe labels are automatically generated by the Bonferroni correction method when comparing the two groups. When the same letter (^a^) is included in the same group, the difference between the two groups is not statistically significant. When ^a^ and ^b^ are included in the same group, the difference between the two groups is statistically significant. ^*^Represents chi-square test.

*¥, RMB per month; EOLC, End of life care; SD, Standard Deviation*.

The outcomes of parental depression revealed differences in both mothers and fathers between both groups (Table 3). The post-natal depression in mothers was significant lower in the family supportive EOLC group compared to mothers in the standard EOLC group (mean 6.90 vs. 7.56; *p* = 0.017). The depression among fathers in the family supportive EOLC group were significantly lower compared to fathers in the standard EOLC group (mean 20.7 vs. 23.1; *p* = 0.001). The outcomes of parent satisfaction revealed differences in that parents in the family supportive EOLC group showed higher satisfaction rates compare to the standard EOLC group (mean 88.9 vs. 86.6; *p* = 0.001).

**Table 3:** Parental depression (mothers and fathers) 1 week after infant's death (*n* = 45).

**Group**	**Cases (*n*)**	**EPDS (mothers)**	**HAMD (fathers)**	**Satisfaction**
Family supportive EOLC (mean and SD)	20	6.90 ± 0.91	20.7 ± 2.05	88.9 ± 1.98
Standard EOLC (mean and SD)	25	7.56 ± 0.87	23.1 ± 2.28	86.6 ± 2.04
t		2.476	3.696	−3.659
*P* value		0.017	<0.001	<0.001

*EOLC, End of life care; EPDS, Edinburgh Postnatal Depression Scale; HAMD, Hamilton Depression rating scale; SD, Standard Deviation*.

## Discussion

To our knowledge, this is the first study to support parents during EOLC in mainland China. The aim of our study was to test a family supportive EOLC intervention to decrease depression among parents and increase parent satisfaction around the death of their infant. The outcome of parent satisfaction with care can be considered an important result. Although no standardized instruments are available to measure satisfaction of EOLC, our hospital questionnaire was sensitive enough to demonstrate differences of overall satisfaction scores between both groups of parents. Further research is needed to develop robust instruments to measure the outcomes of EOLC such as parent satisfaction.

The main cause of death among our included infants was congenital malformations, which is consistent with other studies in China (34, 35). This is in contrast with international studies reporting the main cause of death in neonatology is related to premature birth and infection (36, 37). This difference might be due to the location of our NICU situated in a stand-alone children's hospital. Infants born very premature in other regions of our Hunan province might not have been transferred to our center.

Parental presence during EOLC has been addressed as an important part in neonatal care. The role of the NICU staff in EOLC is to support parents in their mental health and wellbeing as well as empowering parents to take part in the care of their infant during the last days of life (38). The international guideline of FCC in neonatal, pediatric and adult intensive care suggests implementing strategies to improve parental confidence and mental health during and after the NICU (39). In China, the initial steps in implementing FCC in neonatology only started a few years ago (16–18). However, there is limited evidence in FCC practices across the regions in China (40, 41). Our study might contribute to identifying interventions that are feasible and effective in Chinese NICUs who have started recently with FCC practices.

Our study evaluate the family supportive EOLC intervention related to parent depression. In our previous FCC intervention studies we were able to demonstrated improvements in parental depression and anxiety (42, 43). Parents face psychological distress in perinatal and neonatal death with an increased risk of post-traumatic stress disorder, depression, and anxiety. Reports have identified the relationship between perinatal death and the devastating impact on parents, including stress and mental health issues lasting for at least 6 months after the death of their infant (44, 45). During our parent consultation round to discuss our study protocol, parents indicated that they did not want a 6 months follow-up survey. Therefore, we have no follow-up data to inform any long-term support to parents in our community.

In our study, more parents opted for the standard EOLC. Perhaps this can be described to a cultural issue that parents find it difficult in facing the end of life of their infant. A review of Chinese hospice care identified that parents are afraid of staying with their child and experienced more anxiety (46). The perspectives of parents of EOLC in neonatology was explored in a qualitative study among 10 parents (47). These parents indicated that it was extremely important to be able to stay in the NICU regardless the diagnosis on their infant. This “zero separation” has also be addressed as an important issue during the recent two COVID-19 pandemic years (48, 49).

### Limitations

A number of limitations of our study needs to be addressed. First, we used a non-RCT design to provide parents the option to participate in the study. We provided parents the option to choose in what study arm they wanted to participate based on the advice of the parent consultation round before the start of the study. Secondly, the study intervention was not blinded which can potentially influence the outcome measures. The third limitation is that the study was performed at a single center with a small sample size limiting the generalizability of the results for clinical practice. Forth, the different instruments to measure depression among fathers and mothers limited the comparison between the parent couples. Finally, our follow-up was 1 week after the infants' death. Further research is needed to explore long-term impact on parents.

## Conclusion

Neonatal death is still one of the major problems threatening the global health. Our study indicated that providing a comfortable environment and supportive care to parents during the final days of life of an infant decrease their depression and increases parent satisfaction. The NICUs in mainland China and beyond might consider to involve parents in EOLC by providing a single room, have a dedicated psychologist available and provide supportive commemoration materials for parents such as a “yuan man” box.

## Data Availability Statement

The original contributions presented in the study are included in the article/supplementary material, further inquiries can be directed to the corresponding author.

## Ethics Statement

The studies involving human participants were reviewed and approved by Ethics Committee of Hunan Children's Hospital (HCHLL-2020-23). Written informed consent to participate in this study was provided by the participants' legal guardian/next of kin.

## Author Contributions

L-hZ, RZ, X-mP, Y-eX, and JML: concept and design. M-hC, NZ, QT, K-lC, and RZ: data collection. QT, NZ, and RZ: statistical analysis. RZ and JML: drafting of the manuscript. L-hZ, X-mP, Y-eX, NZ, and QT: providing revisions of the manuscript for important intellectual content. All authors contributed to the article and approved the submitted version.

## Funding

The Chinese Nursing Association (number 202028) and the Hunan Children's Hospital Research Foundation (number 202114) financially supported this work.

## Conflict of Interest

The authors declare that the research was conducted in the absence of any commercial or financial relationships that could be construed as a potential conflict of interest.

## Publisher's Note

All claims expressed in this article are solely those of the authors and do not necessarily represent those of their affiliated organizations, or those of the publisher, the editors and the reviewers. Any product that may be evaluated in this article, or claim that may be made by its manufacturer, is not guaranteed or endorsed by the publisher.
